# Supplementary material for: Toxic Psychosocial Stress, Resiliency Resources and Time to Dementia Diagnosis in a Nationally Representative Sample of Older Americans in the Health and Retirement Study from 2006–2016
Source: Int J Environ Res Public Health. 2022 Feb 19;19(4):2419. doi: 10.3390/ijerph19042419 (PMC8875619; doi:10.3390/ijerph19042419)
Supplement: Supplementary file 1 [file ijerph-19-02419-s001.zip › ijerph-1583774-supplementary.pdf]

Supplementary Tables

Table S1. Demographic characteristics of older Americans enrolled in the HRS 2006-2016 sample at baseline by race/ ethnicity.

Characteristic	All (N=6516)	White/ Caucasian (N=5440)	Black / African American (N=844)	Other (N=232)	
	N (%)	N (%)	N (%)	N (%)	p-value
**Age: mean (SD)**	67.5 (7.2)	67.7 (7.3)	66.7 (7.0)	65.6 (6.9)	
**Age categories (years)**					< 0.0001
<=60	1137 (17.5)	927 (17.0)	147 (17.4)	63 (27.2)	
61-70	3288 (50.5)	2693 (49.5)	478 (56.6)	117 (50.4)	
71-79	1772 (27.1)	1531 (28.1)	194 (23.0)	47 (20.3)	
>80	319 (4.9)	289 (5.3)	25 (3.0)	5 (2.1)	
**Sex**					< 0.0001
Male	2396 (36.8)	2058 (37.8)	247 (29.3)	91 (39.2)	
Female	4120 (63.2)	3382 (62.2)	597 (70.7)	141 (60.8)	
**Marital Status**					< 0.0001
Never married	160 (2.5)	105 (1.9)	47 (5.6)	8 (3.4)	
Married/ partnered	4590 (70.4)	4012 (73.7)	416 (49.3)	162 (69.8)	
Separated/Divorced	659 (10.1)	474 (8.7)	160 (19.0)	25 (10.8)	
Widowed	1107 (17.0)	849 (15.6)	221 (26.1)	37 (16.0)	
**Education**					< 0.0001
Less than High School/GED	1452 (22.3)	1038 (19.1)	317 (37.6)	97 (41.8)	
High-school graduate	2140 (32.8)	1828 (33.6)	256 (30.4)	56 (24.1)	
Some college and above	2923 (44.9)	2574 (47.3)	270 (32.0)	79 (34.1)	
**Ever smoked**					0.9474
Yes	3532 (54.7)	2955 (54.8)	453 (54.2)	124 (54.6)	
No	2924 (45.3)	2438 (45.2)	383 (45.8)	103 (46.4)	
**Current alcohol use**					< 0.0001
Yes	3525 (54.1)	3145 (57.8)	301 (35.7)	79 (34.0)	
No	2991 (45.9)	2295 (42.2)	543 (64.3)	153 (66.0)	
**BMI**					< 0.0001
BMI < 18.5 kg/m^2 (Underweight)	49 (0.8)	44 (0.8)	4 (0.5)	1 (0.4)	
BMI 18.5-24 kg/m^2 (Normal weight)	1715(26.6)	1521 (28.3)	128 (15.3)	66 (28.8)	
BMI 25-29 kg/m^2 (Overweight)	2593 (40.3)	2206 (41.1)	298 (35.7)	89 (38.9)	
BMI ≥30 kg/m^2 (Obese)	2080 (32.3)	1601 (29.8)	406 (48.5)	73 (31.9)	
**No. of comorbidities ever had**					< 0.0001
None	969 (14.9)	835 (15.3)	91 (10.8)	43 (18.5)	
One	1809 (27.8)	1549 (28.5)	186 (22.0)	74 (31.9)	
Two	1956 (30.0)	1631 (30.0)	270 (32.0)	55 (23.7)	
Three or more	1782 (27.3)	1425 (26.2)	297 (35.2)	60 (25.9)	
**Retirement Status**					< 0.0001
Not retired	2872 (44.1)	2451 (45.0)	348 (41.2)	73 (31.5)	
Retired plus another status	499 (7.7)	422 (7.8)	63 (7.5)	14 (6.0)	
Completely retired	3144 (48.2)	2566 (47.2)	433 (51.3)	145 (62.5)	
**Moderate physical activity**					< 0.0001
Never	726 (11.1)	629 (11.6)	67 (7.9)	30 (12.9)	
1-4 times per month	3238 (49.7)	2774 (51.0)	359 (42.5)	105 (45.3)	
> 1 time a week	1554 (23.9)	1259 (23.1)	240 (28.4)	55 (23.7)	
Every Day	997 (15.3)	777 (14.3)	178 (21.1)	42 (18.1)	
**Suffer from HD, T2DM or Stroke**					< 0.0001
No	4370 (67.1)	3718 (68.4)	518 (61.4)	134 (57.8)	
Yes	2146 (32.9)	1722 (31.7)	326 (38.6)	98 (42.2)	
**Ever had High blood pressure**					< 0.0001

No	3010 (46.2)	2655 (48.8)	232 (27.5)	123 (53.0)	
Yes	3502 (53.8)	2782 (51.2)	611 (72.5)	109 (47.0)	
**Ever had Stroke**					0.2625
No	6206 (95.3)	5190 (95.5)	795 (94.2)	221 (95.3)	
Yes	306 (4.7)	246 (4.5)	49 (5.8)	11 (4.7)	
**Ever had Diabetes**					< 0.0001
No	5443 (83.6)	4642 (85.4)	634 (75.1)	167 (72.0)	
Yes	1068 (16.4)	793 (14.6)	210 (24.9)	65 (28.0)	
**Ever had Heart problems**					0.4756
No	5326 (81.8)	4434 (81.6)	701 (83.3)	191 (82.7)	
Yes	1182 (18.2)	1001 (18.4)	141 (16.7)	40 (17.3)	

Table S2. Other factors in relation to risk for incident dementia among older adults from HRS 2006-2016.

Characteristic	n/N	Unadjusted HR (95%CI)	p-value	Adjusted HR[c] (95%CI)	p-value
**Demographic Characteristics**					
**Sex: Male vs Female**	133/2396	1.24 (0.95, 1.62)	0.1178	1.21 (0.88, 1.67)	0.2443
**Education**					
Less than High School vs College and above	123/1452	**1.81 (1.34, 2.44)**	**0.0002**	**1.52 (1.09, 2.11)**	**0.0137**
High School vs College and above	93/2140	1.02 (0.75, 1.38)	0.9036	1.01 (0.75, 1.35)	0.9663
**Marital Status**					
Never married vs Married	6/160	1.24 (0.52, 2.98)	0.6208	1.11 (0.44, 2.82)	0.822
Separated/divorced vs Married	31/659	0.90 (0.63, 1.32)	0.6067	0.79 (0.53, 1.17)	0.2286
Widowed vs Married	89/1107	0.76 (0.55, 1.06)	0.1075	0.73 (0.51, 1.05)	0.0871
**Body Mass Index**					
Underweight vs normal	1/49	0.51 (0.06, 4.06)	0.5165	0.56 (0.07, 4.51)	0.5754
Overweight vs normal	117/2593	0.77 (0.55, 1.08)	0.1226	0.71 (0.51, 1.00)	0.0492
Obese vs normal	95/2080	0.96 (0.70, 1.32)	0.8106	0.80 (0.57, 1.13)	0.1955
**Alcohol consumption: No vs Yes**	151/3525	**1.38 (1.06, 1.79)**	**0.0176**	**1.33 (1.01, 1.74)**	**0.0404**
**Cigarette smoking: Yes vs No**	142/4148	**1.48 (1.07, 2.05)**	**0.0182**	**1.32 (1.01, 1.73)**	**0.0418**
**Moderate physical activity**					
1-4 times per month vs Never	163/3238	1.22 (0.79, 1.88)	0.3706	1.26 (0.81, 1.98)	0.3015
> 1 time a week vs Never	73/1554	1.21 (0.76, 1.92)	0.4074	1.14 (0.72, 1.82)	0.5731
Everyday vs Never	72/997	1.37 (0.78, 2.41)	0.2065	1.23 (0.67, 2.27)	0.4935
**Retirement Status**					
Semi-retired vs Not retired	35/499	**2.02 (1.30, 3.13)**	**0.0023**	**2.00 (1.27, 3.16)**	**0.0033**
Completely retired vs Not retired	123/3144	**1.40 (1.10, 1.79)**	**0.0071**	**1.41 (1.10, 1.81)**	**0.0081**
**Health conditions**					
**Comorbid HD, Diabetes/ Stroke: Yes vs No**	151/2146	**1.36 (1.03, 1.79)**	**0.031**	1.31 (0.98, 1.73)	0.0645
**Ever had High blood pressure: Yes vs No**	206/3502	1.25 (0.92, 1.69)	0.1544	1.28 (0.91, 1.81)	0.1522
**Ever had Heart disease: Yes vs No**	91/1182	1.28 (0.94, 1.73)	0.1131	1.31 (0.94, 1.84)	0.1069
**Ever had Diabetes: Yes vs No**	71/1068	1.34 (0.99, 1.81)	0.0576	1.15 (0.83, 1.60)	0.3867
**Ever had Stroke: Yes vs No**	38/306	**2.14 (1.44, 3.16)**	**0.0003**	**2.04 (1.38, 3.02)**	**0.0006**

Note: OR (95%CI): Odds Ratios (95% Confidence Intervals); Bold indicates p-value<0.05; All models adjust for the complex sampling design of the HRS; [c] Adjusted models control for race, toxic stress, and demographic factors; sex, education, alcohol consumption, smoking, BMI, moderate physical activity, retirement status and comorbidity due to Diabetes, Heart diseases and Stroke; Measures of toxic stress and resilience were not mutually adjusted for one another in multivariable models.
